# Increased light scatter in simulated cataracts degrades speed perception

**DOI:** 10.1167/jov.24.13.12

**Published:** 2024-12-20

**Authors:** Samantha L. Strong, Ayah I. Al-Rababah, Leon N. Davies

**Affiliations:** 1 School of Optometry, Aston University, Birmingham, UK

**Keywords:** cataract, light scatter, speed, motion perception, psychophysics

## Abstract

Changes in contrast and blur affect speed perception, raising the question of whether natural changes in the eye (e.g., cataract) that induce light scatter may affect motion perception. This study investigated whether light scatter, similar to that present in a cataractous eye, could have deleterious effects on speed perception. Experiment 1: Participants (n = 14) completed a speed discrimination task using random dot kinematograms. The just-noticeable difference was calculated for two reference speeds (slow; fast) and two directions (translational; radial). Light scatter was induced with filters across four levels: baseline, mild, moderate, severe. Repeated measures analyses of variance (ANOVAs) found significant main effects of scatter on speed discrimination for radial motion (slow *F*(3, 39) = 7.33, *p* < 0.01; fast *F*(3, 39) = 4.80, *p* < 0.01). Discrimination was attenuated for moderate (slow *p* = 0.021) and severe (slow *p* = 0.024; fast *p* = 0.017) scatter. No effect was found for translational motion. Experiment 2: Participants (n = 14) completed a time-to-contact experiment for three speeds (slow, moderate, fast). Light scatter was induced as Experiment 1. Results show increasing scatter led to perceptual slowing. Repeated measures ANOVAs revealed that moderate (*F*(3, 39) = 3.57, *p* = 0.023) and fast (*F*(1.42, 18.48) = 5.63, *p* = 0.020) speeds were affected by the increasing light scatter. Overall, speed discrimination is attenuated by increasing light scatter, which seems to be driven by a perceptual slowing of stimuli.

## Introduction

Rapid, accurate motion perception is crucial for several day-to-day tasks such as: interacting with objects (Kleinschmidt et al., 2002), driving a vehicle (Goodale & Milner, 1992; Anderson & Holliday, 1995; Walter et al., 2001), or moving safely through the environment (Lappe, Bremmer, & van den Berg, 1999; Warren & Rushton, 2009). For example, we need to be able to understand our own (self) movement, relative to the movement of objects around us, which requires complex processing and parsing of retinal image information. This is achievable through complex, parallel processing mechanisms linked to magnocellular pathways and highly specialized motion-sensitive areas in the brain (Merigan, Byrne, & Maunsell, 1991), which not only process the dynamic information reasonably accurately, but also quickly, so that we can make use of it almost automatically. However, unlike with spatial vision, our conscious mind receives little detail about temporal (motion) vision, so it becomes difficult to become aware of changes in ability. That is to say, if an individual's ametropia worsened, they would consciously recognize an increase in blur; however, if their motion perception deteriorated equivalently, it is unlikely they would notice a difference. This differentiation between using information (i.e., recognizing moving objects and avoiding them safely) and being aware of temporal processing (i.e. noticing changes) is important, because impaired motion perception could have a deleterious impact on quality of life, and potentially the safety of the individual and those around them (Hills, 1980).

Linked to this, it is known that motion perception alters as we age. Indeed, several studies have demonstrated that increasing age might affect continuous motion (Norman, Ross, Hawkes, & Long, 2003), motion coherence thresholds (Trick & Silverman, 1991; Gilmore, Wenk, Naylor, & Stuve, 1992; Andersen & Atchley, 1995; Bennett, Sekuler, & Sekuler, 2007; Billino, Bremmer, & Gegenfurtner, 2008; Conlon & Herkes, 2008; Bower & Andersen, 2012), and speed discrimination (Snowden & Kavanagh, 2006). Moreover, researchers have found that the preferred speed of neurons in non-human primates is reportedly lowered, thereby reducing sensitivity to faster speeds (Yang et al., 2008). In contrast, studies have reported no significant effect of age across motion perception tasks (Brown & Bowman, 1987; Mapstone, Dickerson, & Duffy, 2008; Kavcic, Vaughn, & Duffy, 2011), although, the methodology across these different projects is not standardized. Of particular interest, is that more recent research has identified that age-related changes may be linked to specific components of motion perception, which may explain why findings appear inconsistent. Recent work (Guénot et al., 2023) found that coherence thresholds were larger for older adults (70–90 years) but only for radial motion directions (inwards vs outwards). To the contrary, thresholds for translational motion directions were similar to young controls, and thresholds for rotational motion directions were improved. These researchers also report an effect of speed, in that when the stimuli were very fast (14°/s) the radial motion difference vanished. Overall, it is likely that there are a great deal of inter-individual differences present, meaning the most plausible explanation is that motion perception does change with age but to varying extents depending on the individual. Overall, this suggests that the perception of particular types of motion in an older population may be attenuated, highlighting the need for careful exploration into these deficits.

In addition to the impact of healthy ageing on veridical motion perception, there have been mixed reports showing that features such as contrast sensitivity and blur can affect speed perception (Thompson, 1982; Stone & Thompson, 1992; Ascher & Grzywacz, 2000). When contrast is reduced across the whole visual field, speed of external objects appears to be increased (Anstis, 2004; Thompson, Brooks, & Hammett, 2006). Studies simulating the “reduced distance contrast” perceived during foggy conditions found that this led to overestimation of personal speed, leading to slower driving speed (Pretto, Bresciani, Rainer, & Bülthoff, 2012). Furthermore, blurring of a stimulus/object has been shown to decrease motion sensitivity (Leibowitz, Johnson, & Isabelle, 1972). Collectively, these data suggest that an individual with either reduced contrast sensitivity or increased blur would potentially perceive motion inaccurately, which, in turn, could lead to behavioral compensation that may impact their safety (e.g. modified driving speed; see Pretto et al., 2012). Importantly, a cohort who fit these criteria are those who have acquired age-related cataracts. Consequently, given the hypothesis that a reduction in contrast and an increase in retinal blur can affect motion perception, patients with cataracts may experience altered perception of the speed of objects. This is further supported by findings suggesting that motion thresholds improve in children with congenital cataracts after the cataract is treated (Raja et al., 2019). Additionally, and perhaps of most clinical significance, these older patients would be symptomatic, unable to recognize temporal changes to vision. This highlights that it is important to identify whether changes in motion perception do occur, and if so, how severe or impactful they are to vision.

To investigate this, two experiments were designed to test the impact of light scatter on speed discrimination (Experiment 1) and speed perception (Experiment 2). This was achieved by simulating the light scatter of cataracts in a healthy, young population to see if cataracts may be likely to induce any changes in speed perception, while being able to control the severity of the simulated scatter across participants. It was hypothesized that increasing severity of the light scatter (and thereby increasing the severity of the simulated cataract) would decrease sensitivity to speed.

## Experiment 1—Speed discrimination

### Methods

#### Participants

Fourteen participants (control group; P1–P14) were recruited from staff and students at Aston University (age range 20–44 years; median 26.5 years; 10 female, two of whom are authors; vision data can be found in Table 1). Two participants with cataracts (cataract group; C1–C2) were also recruited to be able to compare performances in the simulated conditions to that of genuine cataracts (age range 51–70 years; two female). The participants with cataracts were volunteer-sampled, and presence of cataracts was confirmed by an optometrist using indirect ophthalmoscopy. No participants had any history of contraindicating ocular, neurological, or psychiatric conditions. Experiments were conducted in accordance with the Declaration of Helsinki and were approved by Aston University's Ethics Committee.

**Table 1.** Best-corrected distance visual acuity (VA) and average log contrast sensitivity (CS) for participants who took part in Experiment 1. *Notes*: Data are recorded from the right eye in all cases with the left eye occluded.

	Distance VA (logMAR)	Log CS
P1	−0.18	1.65
P2	−0.10	1.80
P3	−0.22	1.95
P4	−0.10	1.80
P5	−0.20	1.50
P6	0.00	1.95
P7	−0.10	1.95
P8	−0.18	1.95
P9	−0.20	1.80
P10	−0.00	1.80
P11	−0.10	1.95
P12	−0.12	1.50
P13	−0.20	1.95
P14	−0.16	1.95
C1	0.2	1.50
C2	−0.2	1.50

#### Vision and contrast sensitivity

All participants had their distance visual acuity (VA) measured at 3 m using ETDRS presented on a Rexxam LCD 1000-P test chart (Grafton Optical). Contrast sensitivity was measured at 1 m using a Pelli-Robson chart. Vision and contrast sensitivity data are presented in Table 1. For all measures, participants were rendered functionally emmetropic. If they required corrective lenses, they wore their habitual refractive correction (i.e. spectacles or contact lenses).

#### Stimuli and procedure

Stimuli were coded using Psychophysics Toolbox Version 3 (Brainard, 1997; Pelli, 1997; Kleiner et al., 2007) in 32-Bit MATLAB (The MathWorks Inc., Natick, MA), and were displayed on a high-resolution Lenovo Think-Vision monitor with a refresh rate of 60 Hz (Lenovo Group Limited). Mixed black and white random dot kinematograms (RDKs; total 500 dots; each dot subtended 0.2° visual angle within a 12°ø aperture) were presented on a gray background, with the center of the aperture aligned with fixation in the center of the monitor. In control experiments, it was found that there was no difference between unidirectional (all trials the same direction) and bidirectional (direction chosen at random on each trial) presentation, indicating no unintended effects of after-effects or eye movements (see Discussion for more on this). Two directions of motion stimulus were utilized: (1) translational (planar) motion—where all the dots moved upwards on every trial, and (2) radial (global/complex) motion – where all the dots expanded outward from the center on every trial. For the radial motion paradigm, the central 1° ø of the aperture never contained any dots to avoid a confluence indicator at the fixation point. All dots were also assigned a limited lifetime of 20 frames, and were “born” with a random age, to limit the potential for participants to attend to individual dots, or break fixation and follow individual dots with their eyes.

**Figure 1.**
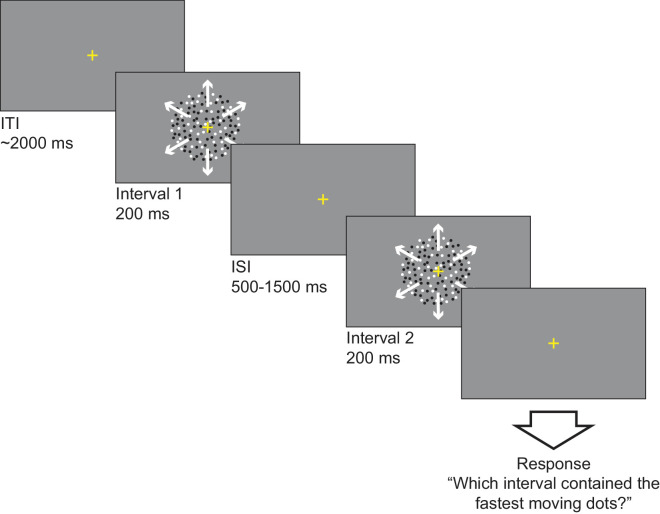
Diagram illustrating the procedure for Experiment 1 using radial motion as an example stimulus for the presented trial. In the diagram, motion direction is indicated by the white arrows.

Participants completed a two-interval forced choice (2IFC) task, where on every trial, two RDKs were presented in sequence within one of the two possible test directions, and in each block the test direction was consistent throughout (see Figure 1). Dots were always shown for 200 ms, with an inter-stimulus interval of between 500–1500 ms (normally distributed around 1000 ms). On every trial, one of the intervals (chosen at random) contained the reference stimulus which moved at the reference speed for that condition. This reference speed was either 3°/s (slow) or 8°/s (fast). The other interval would move at one of seven possible ‘test’ speeds centered on the reference speed. The range of test speeds was designated by the participants’ step size – with three test speeds slower than the reference, three test speeds faster than the reference, and one identical to the reference. Most participants (12/14) used a step size of 15%, whereas two required a larger step size (20%) to obtain a measure of their just-noticeable difference (JND). The task required participants to use the corresponding keys on the computer keyboard to indicate which interval contained the faster moving dots. Each block took approximately six minutes to complete, and participants repeated each block twice to get 30 total repeats for each presented test speed. All conditions were counter-balanced pseudo-randomly across participants to avoid any confounding effects of practice or fatigue.

All conditions were run single-blind, and participants sat 57 cm away from the monitor, which was maintained with a chin and forehead rest. All stimuli were viewed monocularly.

All simulated levels of scatter for the control group were achieved using professional light scattering filters (Hoya 55 mm Fog-A and Hoya 55 mm Fog-B) in order to replicate the phase aberrations present in a cataract (Zuckerman, Miller, Dyes, & Keller, 1973; de Wit, Franssen, Coppens, & van den Berg, 2006). These filters induce forward scatter, which is most similar to the aberrations observed in cortical cataracts (van den Berg, 2018). Using filters in this way has been shown to be an effective way to mimic the straylight characteristics of cataracts, as they induce an appropriate amount of straylight scatter, contrast sensitivity loss, and impact on visual acuity as is observed in a cataract patient (de Wit et al., 2006). Similarly, de Wit et al. (2006) have shown that ‘stacking’ the filters is a reliable way of increasing simulated cataract density. For each task, there were four experimental conditions: Baseline (no filter in place), Mild (1x Hoya Fog-A), Moderate (1x Hoya Fog-B), and Severe (2x Hoya Fog-B). A validation test comparing VA and contrast sensitivity to those previously reported for cataract patients (Elliott, Gilchrist, & Whitaker, 1989) confirmed that these filters were producing similar effects on contrast sensitivity (Table 2). It should also be noted that although the attenuation in VA did not reach the same level as the Grade 3 cataract, this may be due to the age difference between the participants included in the validation (average age 35 years) relative to the cataract patients from the Elliott et al study (average age 69.5 years), as it is known that VA is likely to deteriorate as we age (see Lord, Clark, & Webster, 1991).

**Table 2.** Distance visual acuity (VA) and log contrast sensitivity (CS) for three representative participants to compare the differences across the four scatter simulation conditions (baseline-severe). *Notes*: Data are also compared to that of grade 3 cortical cataract patients from Elliott, Gilchrist and Whitaker (1989). (SD = standard deviation).

	This study (n = 3)					
	Baseline	Mild	Moderate	Severe	Range	Elliott et al. (1989)Grade 3 cortical cataract
VA (logMAR)						
P1	−0.14	−0.10	−0.06	0.06	0.20	
P3	−0.22	−0.14	−0.02	0.10	0.32	
P14	−0.16	−0.10	−0.10	0.06	0.22	
Average	−0.17	−0.11	−0.06	0.05		0.25
SD	±0.04	±0.02	±0.04	±0.02		
Min	−0.14	−0.10	−0.02	0.10		
Max	−0.22	−0.14	−0.10	0.06		
Log CS						
P1	2.00	1.90	1.65	1.20	0.80	
P3	1.90	1.80	1.65	1.15	0.75	
P14	2.00	1.90	1.65	1.25	0.75	
Average	1.97	1.87	1.65	1.20		1.6–1.8
SD	±0.06	±0.06	±0.00	±0.05		
Min	1.90	1.80	1.65	1.15		
Max	2.00	1.90	1.65	1.25		

#### Statistical analyses

For each direction (translational or radial) and reference speed (slow or fast), participants’ data were fitted with a 2-parameter logistic function (Strong, Silson, Gouws, Morland, & McKeefry, 2017) to calculate the JND to highlight the smallest change in speed required for participants to discriminate between two different speeds. This JND was calculated by analyzing the points on the x-axis (speed) which corresponded to the points on the curve where participants were correct 75% of the time for fast (0.75 responses indicating the test was faster than the reference) and slow (0.25 responses indicating the test was faster than the reference) speeds. The difference between the two points was then calculated and divided by two to achieve a measure of estimation of how much the stimulus would need to speed up or slow down to “just notice” the difference relative to chance performance (0.5).

For control participants, statistical analysis of the JND values was performed using the SPSS software package (IBM SPSS Statistics 26). In line with statistical guidelines for clinical studies (Armstrong, Davies, Dunne, & Gilmartin, 2011), repeated-measures analyses of variance (ANOVAs) were calculated across all conditions (baseline, mild, moderate, severe) for each of the directions and speeds. When a significant main effect was present, pairwise comparisons were applied to the data set.

### Results

A two-way repeated measures ANOVA highlighted significant main effects of speed across both directions (radial *F*(1, 13) = 137.43, *p* < 0.01, *η*_p_^2^ = 0.91; translational *F*(1, 13) = 46.15, *p* < 0.01, *η*_p_^2^ = 0.78), which meant it was appropriate to analyze the data for the two speeds (slow and fast) separately.

**Figure 2.**
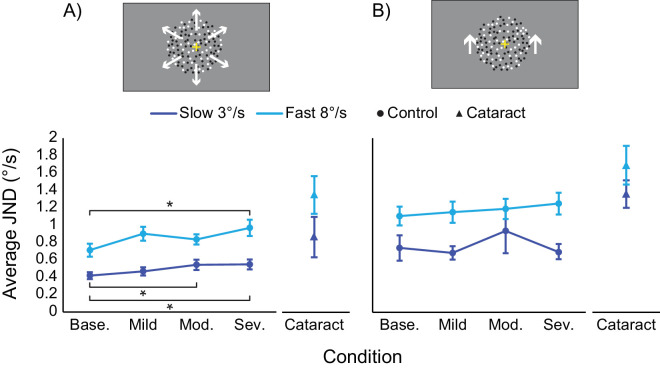
Plots showing the average JNDs for radial (**A**) and translational (**B**) motion conditions for slow (dark blue) and fast (light blue) speeds. Control participants in the simulated scatter condition are shown as circles; participants with cataracts are shown as triangles. For radial motion, increasing the severity of the simulated scatter leads to significant changes in JND for both speeds, whereas no significant difference is found for translational. Error bars represent standard error of the mean. Asterisks represent significant Bonferroni-corrected pairwise comparisons at the 0.05 level (*). (Base – baseline; Mod – moderate; Sev – severe).

From this, a repeated measures ANOVA found significant main effects for radial motion at both speeds (slow *F*(3, 39) = 7.33, *p* < 0.01, *η*_p_^2^ = 0.36; fast *F*(3, 39) = 4.80, *p* < 0.01, *η*_p_^2^ = 0.27). Bonferroni-corrected pairwise comparisons indicate these main effects exist due to increases in JND with the moderate and severe filters (Table 3; Figure 2), suggesting that sensitivity to speed discrimination decreases with increasing light scatter.

**Table 3.** Significance values (Bonferroni-corrected) for each possible pairwise comparison for the radial motion conditions. *Notes*: Statistical significance at *p* < 0.05 is highlighted with an asterisk (*).

	Slow (3°/s)	Fast (8°/s)
Baseline *vs.* Mild	0.082	0.253
Baseline *vs.* Moderate	0.021*	0.596
Baseline *vs.* Severe	0.024*	0.017*
Mild *vs.* Moderate	0.118	1.00
Mild *vs.* Severe	0.290	1.00
Moderate *vs.* Severe	1.00	0.412

No significant main effects were found for translational motion (Figure 2) (slow *F*(3, 39) = 1.36, *p* = 0.269, *η*_p_^2^ = 0.10 ; fast *F*(3, 39) = 1.60, *p* = 0.204, *η*_p_^2^ = 0.11), although data for the cataract patients always appears deteriorated (indicated by higher JNDs across all tasks relative to controls).

Figure 2 also shows that cataract patients appeared to perform better at slow speeds relative to fast speeds, although paired-samples t-tests only reported significant differences for radial motion (*t*(2) = −5.5, *p* = 0.031, *d* = 3.2); no difference was found for translational motion (*t*(2) = −0.8, *p* = 0.499, *d* = 0.47).

In terms of the raw data, it would appear as though performance is worse for fast speeds, as even in the baseline conditions, a larger JND is required to discriminate two fast stimuli, relative to two slow stimuli. However, when these baseline data are converted to Weber fractions, calculated as ratio of threshold discrimination to reference speed (*ΔV/V*), this difference in performance is reversed, with average slow speeds (translational 0.14°/s, radial 0.23 °/s) showing worse performance than average faster speeds (radial 0.09°/s, translational 0.13°/s). This aligns with participant reports and previous literature (Burr, Fiorentini, & Morrone, 1998; Snowden & Kavanagh, 2006) showing that people are typically less sensitive to differences in slow speeds.

To investigate whether increasing age may produce larger speed discrimination deficits in the radial motion task, differences between performance in the baseline task relative to the severe simulation task were calculated to perform a regression.

**Figure 3.**
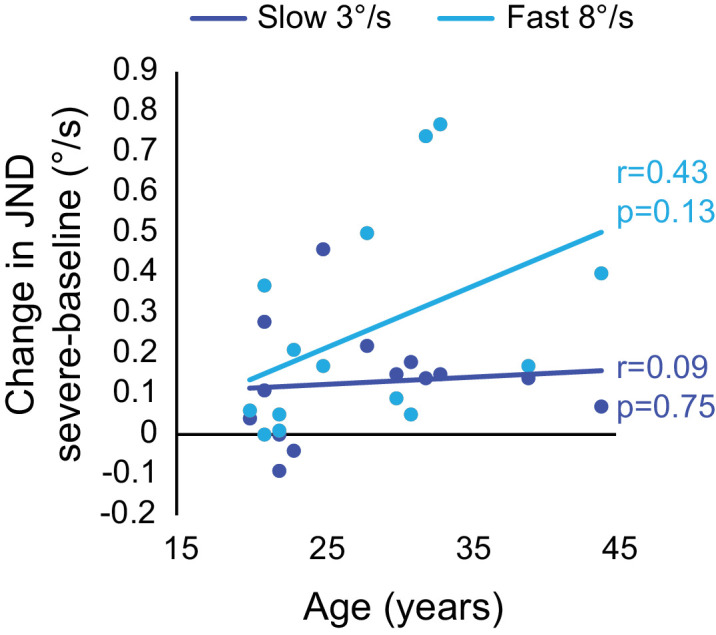
Scatter plot showing the relationship between age (years) and loss of sensitivity (severe JND – baseline JND) for radial motion conditions. Data are plotted for slow (dark blue) and fast (light blue) speeds.

Relationship data between age and loss of sensitivity (Figure 3) for fast speeds were not significantly correlated (*r* = 0.43, *p* = 0.126). There was also no significant correlation for slow speeds (*r* = 0.09, *p* = 0.748).

Taken together, these data suggest that with increasing light scatter (as is expected in development of a cataract), ability to discriminate radial motion speeds may become attenuated (see Discussion for more on this). However, these data do not illuminate the changes that occur with the person's *perception* to lead to this loss of sensitivity. To investigate perceptual changes, it was necessary to run an experiment that directly assessed speed perception, as opposed to discrimination.

## Experiment 2—Speed perception

### Methods

#### Participants

Fourteen participants were recruited from staff and students at Aston University to take part in Experiment 2 (age range 20-44 years; median 22.5 years; 9 female). Twelve of the participants (including two authors) had participated in the previous experiment, whereas two were newly recruited.

#### Vision and contrast sensitivity

Visual acuity, contrast sensitivity, and cataract assessment were all identical to that described in Experiment 1.

#### Stimuli and procedure

**Figure 4.**
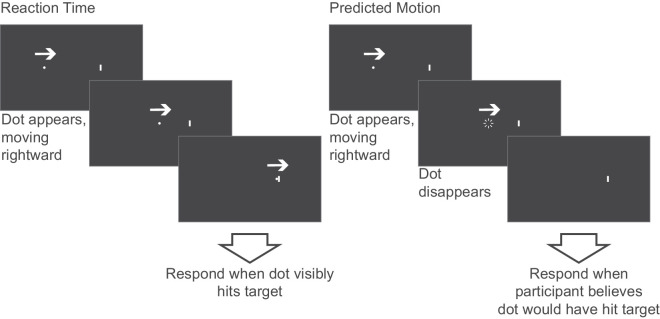
Diagram illustrating the procedure for Experiment 2 using a rightward moving dot as an example stimulus for the presented trial. The left paradigm shows the reaction time task, where the dot remains visible the whole trial. The right paradigm shows the predicted motion task, where the dot disappears either 0.75 s, 1 s, or 1.25 s before it would have hit the target. In the diagram, motion direction is indicated by the white arrows.

Stimuli were coded and presented as described in Experiment 1. This time, however, the stimulus comprised a single white dot (0.2°) on a black background. In the initial block, the white dot appeared randomly at ±10° horizontally from the center of the monitor. The dot then traveled horizontally across the screen towards a vertical white line (target) at one of three speeds (slow 2.5°/s, moderate 5°/s, fast 10°/s) chosen to encompass a wide range of possible speeds. The participant viewed and tracked the dot monocularly and was required to push a button on the keyboard when they perceived the dot to hit the target; this allowed a measure of neural lag (reaction time) which defines how long it took the participant to send the motor signal to their hand to push the button once they believed the dot had hit the target (Figure 4, left).

The next set of blocks comprised the main experiment, in which all parameters were the same as in the initial block, but this time the dot would disappear before it hit the target, and this disappearance would occur either 0.75 s, 1 s, or 1.25 s before it was scheduled to hit the target (time-to-contact; TTC) similar to previous work in this field (Peterken, Brown, & Bowman, 1991; Chang & Jazayeri, 2018). Each of the nine possible dot conditions (3 speeds × 3 TTC) was repeated 10 times, totaling 90 trials. The participant's role was to press a button on the keyboard when they perceived that the dot would have hit the target (Figure 4, right). This allowed a measure of the perceived speed of the stimulus, as it was possible to measure the error (in seconds) between the button press and the assigned TTC. For example, if a person pressed after 0.9 s for a 1 s TTC (−0.1 s error), then they responded to indicate they thought the dot was moving faster than it was, whereas if a person pressed after 1.1 s for a 1 s TTC (0.1 s error), then they responded to indicate that they thought the dot was moving slower than it was. To measure these data as accurately as possible in relation to perceptual experience, and therefore perceptual error, rather than reaction time, the average individual reaction time from the initial block (see Table 4) was subtracted from all error values for each condition to calculate a measure of corrected response (e.g., for a participant with an average reaction time of −0.05 s, this was subtracted from all response times in the TTC tasks). However, although the corrected response is a measure of perceptual error, it only conveys the error in seconds relative to the TTC, and it would be more valuable to understand this error in terms of changes to perceived speed of the dots. To help interpret these values more appropriately, it was possible to convert these initial values into a measure of perceived speed using the equation below, which calculates a ratio of occlusion time (*TTC*) and corrected response (*CR*), multiplied by the genuine dot speed (*v_a_*), to reveal perceived speed (*v_p_*).

$$\begin{array}{c} v_{p}\;=\;\frac{TTC}{CR}\times v_{a} \end{array}$$

**Table 4.** Table showing individual differences in reaction time for when participants perceived the dot to hit the target. *Notes*: P4 and P12 from Experiment 1 did not take part, and so they have been replaced with P15 and P16.

No.	Average reaction time (s)
P1	−0.084
P2	−0.013
P3	0.027
P5	0.022
P6	−0.053
P7	−0.017
P8	−0.004
P9	−0.098
P10	−0.001
P11	−0.115
P13	−0.032
P14	0.000
P15	−0.084
P16	0.008

It is clear from this equation that slower (therefore larger) corrected responses were indicative of slower perceived speeds. This conversion was computed for every trial so that an average perceived speed for each of the three presented speeds could be computed. These numbers were then converted into a measure of error through subtracting the genuine dot speed from the perceived speed to allow consistent comparisons across all presented speeds.

Each of the three possible speeds were presented 30 times, with 10 of each repetition assigned to one of the three possible TTCs. Blocks took approximately five minutes to run, and all conditions were counterbalanced.

#### Statistical analyses

Statistical analyses were performed using the SPSS software package (IBM SPSS Statistics 26). Repeated-measures ANOVAs were calculated across all conditions (baseline, mild, moderate, severe) for each of the speeds (Armstrong et al., 2011). When a significant interaction was found, simple main effects were calculated, and all pairwise comparisons were Bonferroni-corrected for multiple comparisons. Where Mauchley's assumption of sphericity was violated, Greenhouse-Geisser corrections were applied and epsilon (*ε*) values reported.

## Results

**Figure 5.**
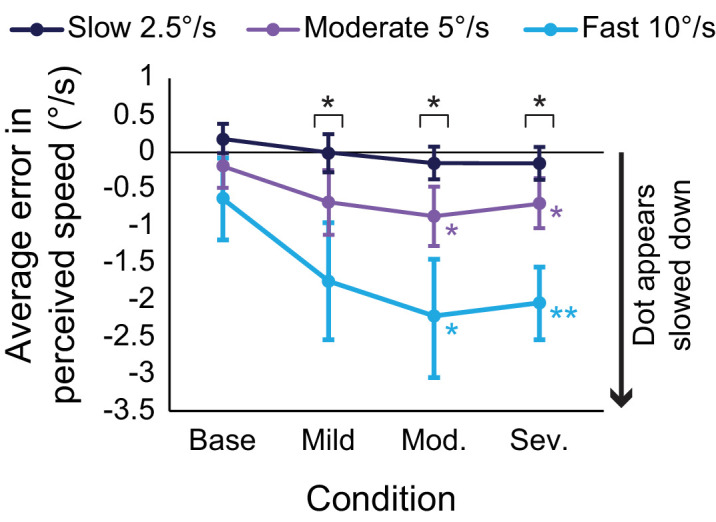
Scatter plot showing the average error in perceived speed for slow (dark blue), moderate (purple), and fast (light blue) speeds. Overall, increasing the severity of the scatter leads to significant changes in error for moderate and fast speeds, whereas no significant difference is found for slow speeds. Color-coded asterisks represent significant Bonferroni-corrected pairwise comparisons showing significant differences in that highlighted condition relative to baseline performance at the 0.05 (*) and 0.01 level (**). Statistical differences were also found between speeds within each of the light scatter conditions (black asterisks). Error bars represent standard error of the mean.

The majority of average errors in perceived speeds were negative, indicating that participants tended to underestimate the speed of the dot (Figure 5), although there was a lot of variance across individuals (see error bars in Figure 5), and it is also clear that the different speeds (slow, moderate, fast) seemed to be affected differently. A two-way repeated-measures ANOVA highlighted a significant interaction between presented speed and light scatter condition (*F*(1.98, 25.72) = 4.66, *p* = 0.019, *ε* = 0.330, *η*_p_^2^ = 0.26). Because of this significant interaction, this means it was inappropriate to perform the standard tests of main effects, and so simple main effects were performed on the data.

Simple main effects do not identify a difference in performance across speeds for the baseline (no light scatter) condition (*F*(1.12, 14.60) = 3.50, *p* = 0.078, *ε* = 0.561, *η*_p_^2^ = 0.21). However, significant differences of error were found for the mild level of scatter across the different presented speeds (*F*(1.02, 13.30) = 10.59, *p* = 0.006, *ε* = 0.512, *η*_p_^2^ = 0.45). Bonferroni-corrected pairwise comparisons reveal this was driven by statistically significant differences between all presented speeds (2.5°/s vs. 5°/s, *p* = 0.012; 2.5°/s vs. 10°/s, *p* = 0.017; 5°/s vs. 10°/s, *p* = 0.026). The same effect of scatter was also reported for the moderate level of scatter (*F*(1.02, 13.24) = 16.73, *p* < 0.001, *ε* = 0.509, *η*_p_^2^ = 0.56), driven by statistically significant differences between all presented speeds (2.5°/s vs. 5°/s, *p* = 0.007; 2.5 vs. 10, *p* = 0.004; 5°/s vs. 10°/s, *p* = 0.003). Similarly, the severe scatter condition also significantly affected performance differently across all speeds (*F*(1.40, 18.17) = 28.01, *p* < 0.001, *ε* = 0.699, *η*_p_^2^ = 0.68), driven by statistically significant differences between all presented speeds (2.5°/s vs. 5°/s, *p* = 0.022; 2.5°/s vs. 10°/s, *p* < 0.001; 5°/s vs. 10°/s, *p* < 0.001). These comparisons indicate that the presence of light scatter affects speeds differently, which would have real-world implications given that the faster speeds are affected to the largest degree.

To investigate the effects of light scatter on each of the speeds, simple main effects were performed. These tests found no statistically significantly effect of light scatter condition on the slow speed (2.5°/s) stimulus (*F*(1.90, 24.63) = 2.40, *p* = 0.114, *ε* = 0.634, *η*_p_^2^ = 0.16). However, increasing light scatter did significantly affect the perceived speed of the moderate speed (5°/s) stimulus (*F*(3, 39) = 3.57, *p* = 0.023, *η*_p_^2^ = 0.22). Similarly, increasing light scatter was also found to produce statistically significant differences within the fast speed (10°/s) stimulus (*F*(1.42, 18.48) = 5.63, *p* = 0.020, *ε* = 0.474, *η*_p_^2^ = 0.30). For both the moderate and fast speed stimuli, significant main effects were driven by statistically significant differences between baseline performance relative to moderate levels of scatter and severe levels of scatter (see Table 5; Figure 5).

**Table 5.** Significance values (Bonferroni-corrected) for each possible pairwise comparison for the predicted motion task. *Notes*: Statistical significance is highlighted with an asterisk depending on whether significance is reached at the 0.05 (*) or 0.01 (**) level.

	Slow (2.5°/s)	Moderate (5°/s)	Fast (10°/s)
Baseline vs. Mild	N/a	0.712	0.618
Baseline vs. Moderate	N/a	0.031*	0.048*
Baseline vs. Severe	N/a	0.038*	0.002**
Mild vs. Moderate	N/a	1.00	0.577
Mild vs. Severe	N/a	1.00	1.00
Moderate vs. Severe	N/a	1.00	1.00

To provide some real-world context, the average difference in speed perception error between severe scatter and baseline conditions was calculated. For moderate speeds the difference in error was −0.50°/s, and for fast speeds it was −1.42°/s. This means that, on average, in the presence of severe scatter, a 5°/s stimulus was perceived as moving at 4.5°/s, and a 10°/s stimulus was perceived as moving at 8.58°/s. It is not straightforward to convert from degrees per second to miles per hour, but if the difference in speed is converted to a percentage, this difference can be understood as 10.0% reduction for moderate speeds and 14.2% reduction for fast speeds. This can be considered as equivalent to perceiving the speed of a 30 mph car as between 25.7 to 27.0 mph.

## Discussion

The data from Experiment 1 show that with the most severe simulation of light scatter, sensitivity to slow radial speeds decreased significantly by 0.13°/s, and sensitivity to fast radial speeds decreased significantly by 0.26°/s (Figure 2) which suggests in purely ecological terms that sensitivity to faster speeds is degraded to a greater extent than slower speeds, although Weber fractions suggest that the slower speeds are degraded *relatively* more. This indicates that light scatter makes it more difficult to tell objects of similar speeds apart, which could have real-world implications for behaviors that require good perception of changes in speed, for example overtaking cars, judging when it is safe to cross a road, or avoiding objects and people in busy environments. When considering this alongside evidence that older people typically show lower sensitivity to motion signals (Trick & Silverman, 1991; Gilmore et al., 1992; Andersen & Atchley, 1995; Norman et al., 2003; Billino et al., 2008), especially radial signals associated with self-navigation (Guénot et al., 2023), this suggests that individuals with cataracts may experience more severely degraded speed perception, which could impact their daily life. Indeed, when comparing the simulated scatter data to that of the mild cataracts observed in the two cataract participants, it appears as though the development of cataracts does lead to even poorer speed discrimination than found in the most severe simulated scatter condition, although this is likely to be confounded by increased age of the cataract group as well. The next step will be to continue this work with more data from cataract patients and age-matched controls, to further explore the putative degradation in speed perception.

**Figure 6.**
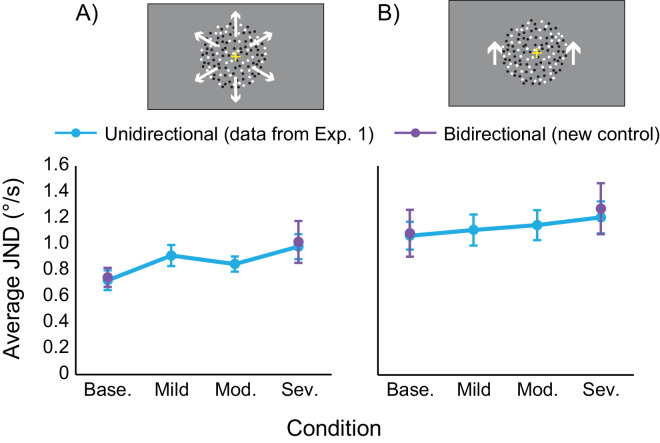
Plots showing the average JNDs for radial (**A**) and translational (**B**) motion conditions for fast speeds across the original data (blue) and control data (purple). Error bars represent standard error of the mean. (Base – baseline; Mod – moderate; Sev – severe).

With experimental paradigms where the direction of motion is defined by 100% coherence as in this experiment, it is possible that motion aftereffects may occur that could potentially lead to unintended differences between conditions through neural adaptation. This is unlikely because the interstimulus interval varied slightly from trial to trial, and the intertrial interval was 2000 ms so it would be unlikely for the short duration of the 200 ms stimulus to induce adaptation effects lasting the duration of the block. However, the presented direction was always consistent within a single run (i.e., upward for translational, outward for radial), and so it was important to confirm experimentally that there was no effect of neural adaptation on the data. To investigate this, a post-hoc control experiment (n = 7) was run where the direction of the stimulus for each trial was chosen randomly within the same type of motion, for example the radial experiment presented either expanding or contracting dots, and the translational experiment presented either upwards or downwards dots. Importantly, direction as consistent between the reference and test within a trial (so as to limit confounding effects of comparisons) and the protocol for this control experiment restricted its scope to only include fast speeds, and only baseline and “severe” scatter conditions because this should reveal differences if there were any. As demonstrated in Figure 6, the direction has no impact on the data, with no difference between the unidirectional and the bidirectional conditions. This therefore provides evidence that the data were not driven by differences in neuronal adaptation.

Another consideration is that it is possible that participants may have tried to use anticipatory eye movements to facilitate their decisions; however, dots were confined to a limited lifetime, which will have restricted the potential for pursuit movements, and if participants were opting to attempt an eye movement strategy, it would be expected to see similar effects across both translational and radial, because dots moved upward vertically from the center in both, albeit in a very narrow range for the radial motion. This similarity across motion types was not observed in the data, which suggests that eye movements cannot explain the results.

Experiment 2 revealed that increasing light scatter can lead to perceived slowing of moderate-fast speeds, which roughly equates to a 10.0% to 14.2% reduction in speed. The increased scatter did not affect slow speeds (2.5°/s), but the data in Figure 6 show the same shape for slow as for moderate and fast speeds. It is, therefore, possible that slow speeds are affected, but the smaller degree of error was not significant given the relatively high degree of interindividual variability. Although this means it will be important to review this in the future, it is also of least clinical relevance, because a perceived slowing of objects at slow speeds (e.g., a person strolling down a corridor) will have much less impact in terms of potentially endangering an individual than a perceived slowing of objects moving at faster speeds (e.g., a car driving on a motorway). Given that these data suggest a significant slowing with increased scatter, it is important that research continues to attempt to quantify this impact to ensure that patient information and safety guidance is as accurate as possible.

**Figure 7.**
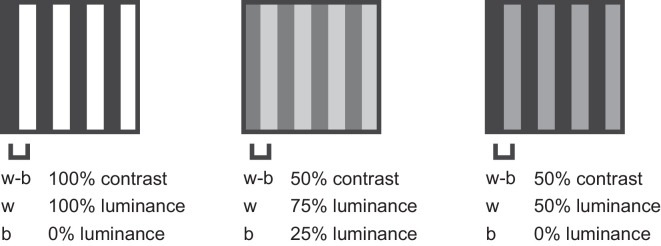
Diagram showing three example gratings, with estimated contrast between white (w) and black (b) stripes defined as 100% (left) or 50% (middle, right). In this hypothetical example, luminance of the stripes is considered as a percentage between 0% (no light) to 100% (as bright as possible).

With regard to previous work, these findings are consistent with literature showing that blurring of an external object will decrease motion sensitivity because the light scatter filters did cause some degree of blur (see VA in Table 2), but it is inconsistent with findings that reducing the contrast leads to increases in perceived speed (Anstis, 2004; Thompson et al., 2006). This is possibly because a reduction in contrast for cataract patients is only part of what happens to their visual experience. Indeed, as light is scattered as it passes through the opacifying crystalline lens, there is also less luminance information reaching the retina, which would affect contrast in a non-linear way. For example, in Figure 7, there is a black and white grating (left) with 100% luminance contrast between stripes (100% luminance defining white stripes and 0% luminance defining black stripes). However, there are also two examples of 50% contrast gratings (middle and right) whereby it is possible to maintain the same luminance contrast with different levels of luminance in the stripes. Previous work has shown that a decrease in contrast might affect speed perception (Anstis, 2004), but in this project the filters contributed to scattering the light—meaning that stimuli with lower luminance would likely be affected to a greater extent than stimuli with greater luminance, although stimuli with higher levels of luminance would likely also produce greater amounts of glare than those with lower luminance. Overall, although cataracts and light scatter do contribute to contrast information, it is likely that the effect on vision is not as simple as being limited to contrast information alone.

There was also a trend in the data for decreases in speed sensitivity to be worse for older participants (>30 years) which aligns with previous work (Trick & Silverman, 1991; Gilmore et al., 1992; Andersen & Atchley, 1995; Norman et al., 2003; Billino et al., 2008; Guénot et al., 2023) showing the impact of healthy ageing on motion sensitivity, but the difference between age groups reported here was not significant so it may be that a larger sample size would be required to conclusively report on age-related effects, or it may be that the age range (20–44 years) was not large enough to yield a measurable difference across groups. This finding also ties in with one of the limitations of this design, in that the median age of participants was very young (∼26–27 years), which does make it likely that their speed sensitivity at baseline was likely lower (better) than it would be for people at an age where they will be developing cataracts. However, this does not undermine the identified significant decrease in sensitivity with increasing scatter, because this was found within-subjects so it would be expected to persist regardless of age–although possibly to different extents. It will be useful therefore to collect more data on age-matched controls in the future, to see how speed perception is affected with age and increasing scatter.

Overall, these data suggest that with increasing light scatter associated with increasing cataract severity, it is likely that there will be a loss of sensitivity to radial motion signals which may be due to a perceived slowing of objects in the environment. This type of motion is important for navigating safely through the environment as it forms a crucial signal in understanding movement of the self, relative to the environment, so this could represent significant difficulties in cataract patients’ lives. It will be important to quantify this further, and determine the relative impact of these effects on cataract patients of different types and varying severity.
